# Hepatitis C in European prisons: a call for an evidence-informed response

**DOI:** 10.1186/1471-2334-14-S6-S17

**Published:** 2014-09-19

**Authors:** Amber Arain, Geert Robaeys, Heino Stöver

**Affiliations:** 1 Limburg Clinical Research Programme, Faculty of Medicine and Life Sciences, Hasselt University, Hasselt, Belgium; 2 Department of Gastroenterology and Hepatology, Ziekenhuis Oost-Limburg, Genk, Belgium; 3 Limburg Clinical Research Programme, Faculty of Medicine and Life Sciences, Hasselt University, Hasselt, Belgium; 4 Department of Hepatology, University Hospitals Leuven, Leuven University, Leuven, Belgium; 5 Faculty of Health and Social Work, University of Applied Sciences, Frankfurt, Germany

## Abstract

Globally, over 10 million people are held in prisons and other places of detention at any given time. People who inject drugs (PWID) comprise 10-48% of male and 30-60% of female prisoners. The spread of hepatitis C in prisons is clearly driven by injection drug use, with many infected prisoners unaware of their infection status. Risk behaviour for acquisition of hepatitis C via common use of injecting equipment is widespread in many prison settings.

In custodial settings, effective and efficient prevention models applied in the community are very rarely implemented. Only approximately 60 out of more than 10,000 prisons worldwide provide needle exchange. Thus, HCV prevention is almost exclusively limited to verbal advice, leaflets and other measures directed to cognitive behavioural change. Although the outcome of HCV antiviral treatment is comparable to non-substance users and substance users out of prison, the uptake for antiviral treatment is extremely low.

Based on a literature review to assess the spread of hepatitis C among prisoners and to learn more about the impact for the prison system, recommendations regarding hepatitis C prevention, screening and treatment in prisons have been formulated in this article.

## Introduction

Globally, more than 10 million people are held in prisons and other places of detention at any given time [1]. Due to the high turnover rate in the prison population, it is estimated that more than 30 million people spend time in prisons each year. Drug users in particular often spend relatively short periods in prisons before returning to their communities.

Many people held in prisons have severe problems associated with drug use, together with related health and social disadvantages. Those categorised as problematic drug users constitute a substantial proportion of prison populations in Europe. Counting only sentenced prisoners with drug offences as the main offence, 15 of 26 European countries for which information is available report proportions over 15% [2]. The number of drug users in prisons is even higher. A systematic review of international studies – with a preponderance of studies conducted in the United States – found that 10% to 48% of men and 30% to 60% of women were dependent on or used illicit drugs in the month before entering prison [3]. In the European Union, it has been estimated that about half of all members of the prison population have used illicit drugs at some time in their lives [4].

Hepatitis C virus (HCV) infection, which is both preventable and treatable, is a major concern in correctional settings. People who inject drugs (PWID) have high rates of imprisonment, largely due to the criminalization of their drug use and to the tendency to fund drug use through crime. The dynamics of illicit drug use, HCV infection and imprisonment are closely intertwined [5]. One study found that in Australian prisons, one-third of entering inmates tested positive for HCV antibodies. The proportion of positive results among entering inmates who injected drugs was 56%. Furthermore, one-third of inmates who were anti-HCV positive were unaware of their infection status.

In general, 80% of HCV-infected individuals develop chronic HCV. Of these, 10% to 15% will develop liver cirrhosis [6]. Three to four percent of patients with cirrhosis develop hepatocellular carcinoma every year [7,8]. Worldwide, 25% of liver cancer cases are attributable to HCV infection [9].

Given the interplay between HCV, drug use and incarceration, HCV has the potential to impose a major disease burden on European prison populations. The purpose of this article is to review evidence and formulate recommendations regarding how to address this situation.

## HCV transmission, risk factors and prevention in prisons

Imprisonment is an independent risk factor for HCV infection for PWID in the community [10-15]. All modes of HCV transmission that occur in the community also occur in prisons. In particular, HBV, HCV and HIV are transmitted in prisons through the sharing of contaminated injecting equipment, and also through unsafe sexual contact, unsafe skin penetration (such as piercing and tattooing, sharing of razors, and blood-sharing rituals) and the improper sterilisation or reuse of medical or dental instruments [16]. Some PWID continue to use drugs such as opioids, including by injection, while incarcerated, and some people initiate injecting in prison [17]. In Australian prisons about half of all imprisoned people who inject drugs continue to inject drugs in prison [18].

One of the most important risk factors for HCV infection is intravenous drug use while in prison [12,15]. A meta-analysis of 30 studies from different countries showed a clear association between the prevalence of HCV infection in prisoners and their history of injecting drug use. There were weaker associations with female gender and with tattooing. The results showed that HCV seroprevalence was approximately 11% higher among already-detained inmates, as opposed to inmates entering prison. A strong association between HCV infection and the length of time spent in prison was also seen. These findings suggest that intra-prison transmission may contribute considerably to high HCV levels in prison populations [7].

The prevalence of HCV infection among prison inmates is many times higher in most custodial settings than in the general population [19,20], primarily because of the high proportion of people who inject drugs (PWID) [6] who are known to be at high risk of infection. Esteban et al concluded that HCV prevalence in the general population in Western Europe is 0.5%, and that it is 2.5% and 6% in Southern Europe and Eastern Europe respectively [21]. A meta-analysis performed by Vescio et al (2008), showed that there is a high HCV prevalence in inmates in several countries around the world. HCV prevalence in inmates was approximately 30% to 40% (range: 2%–58%) [7].

Different studies from Europe, Australia and the United States suggest that hepatitis C prevalence rates in prisons range from 8% to 57% [9,22-24].

In prisoners with a history of injecting drug use, the global summary prevalence was 64% [25]. Data on HCV antibody prevalence among injecting drug users in European prisons between 2005 and 2010 were reported by five countries, with prevalence ranging from 12% in Hungary to 91% in Luxembourg [26]. Among female prisoners the prevalence is two in three. Among female PWID, the prevalence can be even higher, ranging from 49% to 88% [27].

Patterns of hepatitis C prevalence in custodial settings include increasing prevalence with age; higher prevalence among female prisoners; and increasing prevalence with multiple admissions to prisons (AIHW 2010). Infection with more than one strain of HCV may also be common in prison populations; one study found 24% prevalence of multiple infections within a cohort of prisoners who inject drugs [28].

The mortality rate for HCV-induced liver disease in prisons is high. Chronic liver disease-related deaths accounted for 16% of deaths among male Texan prisoners from 1989 to 2003. Either hepatitis B virus (HBV) or HCV has been identified as a causal factor in more than one-third of chronic liver disease-related deaths [29].

## Health care for prison inmates

Prisoners are entitled, without discrimination, to a standard of health care equivalent to that available in the outside community, including preventive measures. This principle of equivalence is fundamental to the promotion of human rights and best health practice within prisons, and is supported by international guidelines on prison health and prisoners’ rights, as well as national prison policy and legislation in many countries [30].

People should not leave custody in a worse condition or with poorer health than when they entered [18]. The period of incarceration should be viewed as a public health window of opportunity, including HCV testing, treatment, care and support [31]. There is consensus among international organisations that all blood-borne virus prevention, treatment and care interventions that are available in the community, including harm reduction interventions, must also be available to prisoners [32-34].

Effective and efficient prevention models that are applied in the community are very rarely implemented in custodial settings. Only about 60 out of more than 10,000 prisons worldwide provide needle exchange [35]. Thus, HCV prevention is almost exclusively limited to verbal advice, leaflets and other measures directed toward cognitive behavioural change. As HCV spreads primarily via injecting drug use in prisons, dependence-driven behaviour can be expected to predominate [36].

## HCV screening in prisons

Many people enter prison with social, medical, and mental health conditions and re-enter the community with few of these conditions having been addressed while incarcerated. Hepatitis C is one such condition, and its management challenges both the correctional and public health systems. Identifying all cases of HCV among inmates is an essential first step, but testing strategies for blood-borne viruses and test coverage vary globally between jurisdictions. In some countries there is no testing procedure at all [37], while some use voluntary screening and others use a targeted approach. This situation suggests a need for ongoing surveillance using a standardized approach to reliably report prevalence. Ideally, surveillance should include collection of data on incident cases [5].

Screening for HCV infection and uptake of antiviral therapy are low in prisons. Uptake for screening ranges between 9% and 24% [38,39]. In a nationwide survey in the United States, only one of 36 states reported routine screening, and only one reported conducting a seroprevalence study in custodial care [6]. Of 3,034 new prisoners at Dartmoor Prison (England), 12% were screened, with 16% of these found to be seropositive. Seventy-nine percent of seropositive prisoners with a positive polymerase chain reaction result were confirmed as cases of positive viremia, and 27% of these prisoners had a biopsy. Two prisoners were eligible for treatment [40].

The results of a recent cost-effectiveness study [41] indicated that the introduction of dried blood spot testing compared to venipuncture for HCV case-finding was likely to be cost-effective in prisoners in the United Kingdom and the United States if a minimum level of continuity of care in treatment or referral between prison and the community could be ensured.

Qualitative research has described barriers to testing such as a lack of proactive approaches to offering testing, prisoners’ fears and lack of knowledge about HCV, low motivation for testing, and concerns about confidentiality and stigma, which may mean fewer people are tested [42,43]. More work is needed to increase the level of testing in prisons.

## HCV treatment for prisoners

With good adherence, HCV treatment outcomes for incarcerated patients who take combination therapy (peg-interferon and ribavirin) are comparable to those observed in non-incarcerated patients at similar stages of disease [44,45]. Studies performed in custodial settings show acceptable results and sustained viral response (SVR) rates ranging between 36% and 66% [44-49].

Rates of HCV treatment completion and SVR observed in correctional populations have been similar to those reported in community samples [44,49]. The re-infection rate after successful antiviral treatment in prisons is low (7%) [50], and is comparable to re-infection rates outside of prisons. Antiviral treatment in prison also appears to be cost-effective according to a modelling study that looked at a US prison population [51].

Several groups have argued that correctional institutions are an important setting for health interventions such as screening, diagnosis, prevention, and treatment of HCV infection [52,53,73]. One of the reasons is that in prison it is possible to monitor patients more closely, and to address side-effects and provide psychiatric care as necessary [54]. A second reason is that prisons provide an opportunity to engage with a difficult-to-reach population – incarceration may be the first or only time that many inmates intersect with the healthcare system. A third reason is that medical management and adherence to antiviral therapy require lifestyle stability, which can be provided by incarceration, particularly for offenders with a history of mental illness or substance abuse [53].

## Programmes developed to improve HCV treatment in prison

In a few studies, intervention programs were developed or tested to improve the management of hepatitis C in prisons. Arora S et al developed Project ECHO, a programme that utilized teleconferencing, videoconferencing, and e-mail communication to connect specialists with primary care providers in prisons and rural areas in order to improve access to quality health care for New Mexicans with hepatitis C. [55]. Through Project ECHO, 226 patients received interferon and ribavirin treatment for hepatitis C.

In the US state of New York, a programme was created to provide continuity of HCV treatment to prisoners upon their release. [56]. A referral process was developed, staff were mobilized, and health-care facilities in the community were recruited to accept referrals. This programme included 70 prisons and 21 health care facilities. Until March 2006, 24 inmates were enrolled.

Another treatment programme was developed in the North Dakota Department of Corrections and Rehabilitation. [57]. The treatment protocol followed National Institutes of Health guidelines for primary therapy for hepatitis C, with the exception of replacing weekly pegylated interferon administration with three-times-weekly consensus interferon administration. The programme resulted in sustained viral responses of 54% for genotype 1, 75% for genotypes 2 and 3, and 64% overall.

Research indicates that nurses play a crucial role in providing education, support and management of patients infected with hepatitis C [58-61]. The involvement of nurses enhances access to treatment, treatment adherence and response to treatment [58,61,62]. A recent study by Lloyd et al (Lloyd et al. 2013) evaluated the safety and effectiveness of a nurse model of care for inmates. In this study, treatment was initiated in 108 patients (28% of the 291 patients enrolled in the study) and the SVR rate among patients with complete follow-up data was 69%. This first prospective treatment programme in a prison setting demonstrated that the nurse-led model of hepatitis C care enhanced treatment uptake and reduced the burden of disease.

## Staff training and support

Staff training and support are important because all people working in prisons should be aware of blood-borne viruses and of the universal and special precautions that are recommended for preventing transmission. Training and support should be tailored to the needs of different types of staff working within and outside of health services. Prison administrators are advised to: [24,63].

• Provide target-group specific peer education and training on hepatitis and other communicable diseases, routes of transmission in the workplace (e.g., the risk of needle-stick injuries occurring during searches of cells), confidentiality, drug use, hepatitis prevention measures, hepatitis testing and treatment opportunities, drug dependence treatment, universal precautions and use of protective equipment, and the rationale for and content of prison rules and policies related to hepatitis to all prison staff as part of their initial training, and update this training on a regular basis during the course of employment.

• Ensure that the training of prison staff addresses hepatitis-related discrimination and homophobia, reduces staff opposition to the provision of hepatitis prevention measures to prisoners, emphasises the importance of confidentiality and non-disclosure of hepatitis status and medical information, and promotes the compassionate treatment of prisoners living with hepatitis. Ensure access to appropriate post-exposure prophylaxis and counselling.

## General recommendations

The following recommendations can be made regarding hepatitis C prevention, screening and treatment in prisons:

• Close collaboration between prison and public (or community) health services is needed (e.g. in order to facilitate community follow-up of treatment; [6,64]. Ensure continued hepatitis C treatment and care when there is movement between custodial settings, and when inmates receiving treatment re-enter the community [7,18,56].

• Incarcerated persons with risk factors for HCV infection should be screened for viral hepatitis infections [65].

• There is a need to develop approaches to increase the uptake of testing by raising awareness amongst prisoners about HCV infection, optimising testing pathways that support appropriate testing at appropriate times during a prisoner's stay in prison, ensuring adequate pre- and post-test discussion, and developing care pathways for HCV that enable seamless continuity of care [42]. Proven nurse-led intervention models could be transferred into the prison setting in order to guarantee guidance.

• Prisoners should be provided with substance abuse treatment. Opiate agonist therapy (methadone, buprenorphine or diacetylmorhpine) should be administered to opiate-dependent subjects with hepatitis B and C infections in order to reduce the risks of transmission and re-infection.

• There is a need to provide sterile injecting equipment and other harm reduction measures to those who inject while in prison [66,67]. HCV-infected persons should be counselled on how to avoid transmitting HCV to others. [68]

• Health education activities (including peer education) should be carried out, in particular for inmates with no or minimal prior health education [69,70].

• Depression and psychosis, which are common in prison settings, occur with interferon treatment. It is essential to provide psychiatric evaluation of patients prior to and during treatment, in order to avoid or control the possible appearance of mental side effects [53,71].

• A multidisciplinary approach through the collaboration of addiction specialists, hepatologists, infectious disease experts, clinical psychologists, nurses and prison physicians should be adopted [72].

• If possible, a directly observed treatment (DOT) strategy, which ensures supervision of oral therapy administration and the injection of subcutaneous therapy by health care professionals, should be used, as occurs in anti-HIV and tuberculosis treatment in prison inmates [19].

## Conclusion

HCV prevalence is very high in prisons. Intravenous drug use while in prison is one of the most important risk factors. The utilization of harm reduction strategies in order to prevent transmission of HCV in prisons lags far behind similar efforts taking place outside of prisons. The scarcity of prison-based needle exchange programmes is a prominent example of this problem. Although testing for HCV in prisons should be a cornerstone in the health care of prison inmates, levels of screening for HCV infection and uptake of antiviral therapy in prisons are low. Since HCV treatment outcomes for incarcerated patients are comparable to those observed in non-incarcerated patient, programmes to improve HCV treatment in prison, staff support and recommendations regarding HCV have been developed and must be implemented. Treatment for HCV in prison should be routinely available and offered under standard guidelines and protocols equivalent to those applied in the community.

## Competing interests

The authors declare that they have no competing interests.

## Declarations

This article has been published as part of *BMC Infectious Diseases* Volume 14 Supplement 6, 2014: Viral Hepatitis in Europe. The full contents of the supplement are available online at http://www.biomedcentral.com/bmcinfectdis/supplements/14/S6. The publication charges for this supplement were funded by AbbVie as an unrestricted grant to Rigshospitalet, the University of Copenhagen. AbbVie further funded the printing of the supplement with additional financial support from the Drug Prevention and Information Programme (DPIP) of the European Union.

## References

[B1] Open Society Foundations Improving Health in Pretrial Detention: Pilot Interventions and the Need for Evaluation 2011 http://www.soros.org/initiatives/health/focus/ihrd/articles_publications/publications/pretrial-detention-health-20110531 Accessed 29 March 2013

[B2] Carpentier C Royuela L Noor A Hedrich D Ten Years of Monitoring Illicit Drug Use in Prison Populations in Europe: Issues and Challenges The Howard League of Criminal Justice 2012 51 1 37 66

[B3] Fazel S Bains P Doll H Substance abuse and dependence in prisoners: a systematic review Addiction 2006 101 2 11 191 10.1111/j.1360-0443.2006.01316.x 16445547

[B4] Zurhold H Haasen C Stöver H Female drug users in European prisons: a European study of prison policies, prison drug services and the women’s perspectives 2005 Oldenburg, D: Bibliotheks- und Informationssystem der Carl von Ossietzky Universität

[B5] Reekie JM Levy MH Richards AH Wake CJ Sidall DA Beasley HM Kumar S Butler TG Trends in prevalence of HIV infection, hepatitis B and hepatitis C among Australian prisoners — 2004, 2007, 2010 MJA 2014 200 5 277 280 24641153 10.5694/mja13.11062

[B6] Spaulding AC Greene C Davidson K Schneidermann M Rich J Hepatitis C in state correctional facilities Preventive Medicine 1999 29 92 100 9973592 10.1006/pmed.1998.0418

[B7] Vescio MF Longo B Babudieri S Starnini G Carbonara S Rezza G Monarca R Correlates of hepatitis C virus seropositivity in prison inmates: a meta-analysis Journal of Epidemiology and Community Health 2008 62 4 305 313 18339822 10.1136/jech.2006.051599

[B8] Macalino GE Vlahov D Sanford-Colby S Patel S Sabin K Salas C Rich JD Prevalence and incidence of HIV, hepatitis B virus, and hepatitis C virus infections among males in Rhode Island prisons American Journal of Public Health 2004 94 7 1218 1223 15226146 10.2105/ajph.94.7.1218 PMC1448424

[B9] Schulte J Wu H Kelley MJ Grady J Linthicum L Dunn K Hepatitis C seroprevalence among newly incarcerated inmates in the Texas correctional system Public Health 2003 117 1 43 48 10.1016/s0033-3506(02)00009-4 12802904

[B10] van Beek I Dwyer R Dore GJ Luo K Kaldor JM Infection with HIV and hepatitis C virus among injecting drug users in a prevention setting: retrospective cohort study British Medical Journal 1998 317 7156 433 437 9703523 10.1136/bmj.317.7156.433 PMC28635

[B11] Butler T Boonwaat L Hailstone S Falconer T Lems P Ginley T Read V Smith N Levy M Dore G Kaldor J The 2004 Australian prison entrants' blood-borne virus and risk behaviour survey Australian and New Zealand journal of public health 2007 31 1 44 50 17333608 10.1111/j.1753-6405.2007.00009.x

[B12] Hellard ME Hocking JS Crofts N The prevalence and the risk behaviours associated with the transmission of hepatitis C virus in Australian correctional facilities Epidemiology and Infection 2004 132 3 409 415 15188710 10.1017/s0950268803001882 PMC2870120

[B13] Hennessey KA Kim AA Griffin V Collins NT Weinbaum CM Sabin K Prevalence of infection with hepatitis B and C viruses and co-infection with HIV in three jails: a case for viral hepatitis prevention in jails in the United States Journal of Urban Health 2009 86 1 93 105 18622707 10.1007/s11524-008-9305-8 PMC2629523

[B14] Miller ER Bi P Ryan P Hepatitis C virus infection in South Australian prisoners: seroprevalence, seroconversion, and risk factors International Journal of Infectious Diseases 2009 13 2 201 208 18790659 10.1016/j.ijid.2008.06.011

[B15] Poulin C Alary M Lambert G Godin G Landry S Gagnon H Demers E Morarescu E Rochefort J Claessens C Prevalence of HIV and hepatitis C virus infections among inmates of Quebec provincial prisons Canadian Medical Association Journal 2007 177 3 252 256 17664448 10.1503/cmaj.060760 PMC1930200

[B16] WHO UNODC UNAIDS Technical Guide for countries to set targets for universal access to HIV prevention, treatment and care for injecting drug users 2009 Geneva, Switzerland http://www.unaids.org/en/media/unaids/contentassets/dataimport/pub/manual/2010/idu_target_setting_guide_en.pdf

[B17] Hedrich D Alves P Farrell M Stöver H Möller Mayet S The effectiveness of opioid maintenance treatment in prison settings: a systematic review Addiction 2012 107 3 501 517 21955033 10.1111/j.1360-0443.2011.03676.x

[B18] Hepatitis Australia Consensus statement: Addressing hepatitis C in Australian custodial settings 2011 http://www.hepatitisaustralia.com/__data/assets/pdf_file/0008/2123/Prisons-consensus-statement.pdf Accessed 30 March 2013

[B19] Haber P Parson S Harper S White PA Rawlinson WD Lloyd AR Transmission of hepatitis C within Australian prisons Medical Journal of Australia 1999 171 1 31 33 10451669 10.5694/j.1326-5377.1999.tb123494.x

[B20] Vlahov D Nelson KE Quinn TC Kending N Prevalence and incidence of hepatitis C virus infection among male prison inmates in Maryland European Journal of Epidemiology 1993 9 5 566 569 8307145 10.1007/BF00209538

[B21] Esteban JI Sauleda S Quer J The changing epidemiology of hepatitis C virus infection in Europe Journal of Hepatology 2008 48 1 148 62 18022726 10.1016/j.jhep.2007.07.033

[B22] Crofts N Hopper JL Milner R Breschkin AM Bowden DS Locarnini SA Blood-borne virus infections among Australian injecting drug users: implications for spread of HIV European Journal of Epidemiology 1994 10 6 687 694 7672048 10.1007/BF01719282

[B23] POST JJ Arain A Lloyd AR Enhancing assessment and treatment of hepatitis C in the custodial setting Clin Infect Dis 2013 57 Suppl 2 S70 4 23884069 10.1093/cid/cit265

[B24] Robaeys G Grebely J Mauss S Bruggmann P Moussalli J De Gottardi A Swan T Arain A Kautz A Stöver H Wedemeyer H Schaefer M Taylor L Backmund M Prins M Dore GJ International Network for Hepatitis in Substance Users Recommendations for the management of hepatitis C virus infection among people who inject drugs Clinical Infectious Diseases 2013 57 Suppl 2 129 137 10.1093/cid/cit302 23884061

[B25] Larney S Kopinski H Beckwith CG Zaller ND Des Jarlais D Hagan H Rich JD van den Bergh BJ Degenhardt L Incidence and Prevalence of Hepatitis C in Prisons and Other Closed Settings: Results of a Systematic Review and Meta-Analysis Hepatology 2013 1215 1224 23504650 10.1002/hep.26387 PMC3723697

[B26] EMCDDA Prisons and Drug sin Europe: The problem and responses Lisbon/Portugal 2012 http://www.emcdda.europa.eu/publications/selected-issues/prison Accessed 30 March 2013

[B27] Viitanen P Vartiainen H Aarnio J von Gruenewaldt V Hakamäki S Lintonen T Mattila AK Wuolijoki T Joukamaa M Hepatitis A, B, C and HIV infections among Finnish female prisoners--young females a risk group Journal of Infection 2011 62 1 59 66 21087630 10.1016/j.jinf.2010.10.011

[B28] Pham ST Bul RA Bennett JM Rawlinson WD Dore GJ Lloyd AR White PA Frequent Multiple Hepatitis C Virus Infections Among Injection Drug Users in a Prison Setting Hepatology 2010 52 1564 1572 21038409 10.1002/hep.23885

[B29] Harzke AJ Baillargeon J Paar DP Pulvino J Murray OJ Chronic liver disease mortality among male prison inmates in Texas, 1989-2003 American Journal of Gastroenterology 2009 104 6 1412 1419 19491854 10.1038/ajg.2009.106 PMC2856927

[B30] WHO Prison Health as Part of Public Health Declaration, Moscow 24 October 2003 2003 http://www.euro.who.int/__data/assets/pdf_file/0007/98971/E94242.pdf

[B31] MACASHH Hepatitis C Prevention, Treatment and Care: Guidelines for Australian Custodial settings evidence base for the Guidelines 2008 http://www.health.gov.au/internet/main/publishing.nsf/content/9F632131D38B580CCA257505007C1A70/$File/prison-guidelines-evidence.pdf Accessed 30 March 2013

[B32] UNAIDS Prisons and AIDS: UNAIDS point of view 1997 Geneva, CH: UNAIDS https://www.unodc.org/documents/hiv-aids/UNAIDS%20prison%20and%20AIDS.pdf

[B33] UNODC WHO UNAIDS HIV/AIDS Prevention, Care, Treatment and Support in Prison Settings. A Framework for an Effective National Response 2006 New York, Vienna & Geneva: United Nations Office on Drugs and Crime, World Health Organization and Joint United Nations Programme on HIV/AIDS http://www.unodc.org/pdf/criminal_justice/HIV-AIDS_Prevention_Care_Treatment_and_Support_in_Prison_Settings.pdf

[B34] WHO WHO guidelines on HIV infection and AIDS in prisons 1993 Geneva, CH: WHO (WHO/GPA/DIR/93.3) https://www.unodc.org/documents/hiv-aids/WHO%20guidelines%20prisons.pdf

[B35] Hariga F Stöver H Guide to starting and managing prison-based needle and syringe programmes PNSP) REVISTA ESPAÑOLA DE SANIDAD PENITENCIARIA. Comunicaciones del IX Congreso de Sanidad Penitenciaria y XVI Jornadas de la SESP, Suplemento 2012 14 S. 35 http://www.lalunadelmediodia.es/wp-content/uploads/2013/01/rspen.pdf

[B36] Stöver H Kastelic A WHO Drug treatment and harm reduction in prisons Health in Prison. A Practical Guide 2014 2 Copenhagen/Denmark 113 133 http://www.euro.who.int/en/health-topics/health-determinants/prisons-and-health/who-health-in-prisons-programme-hipp

[B37] Stöver H Thane K Towards a Continuum of Care in the EU Criminal Justice System A survey of prisoners’ needs in four countries (Estonia, Hungary, Lithuania, Poland) 2011 Oldenburg/Germany: Bis-Verlag

[B38] Skipper C Guy JM Parkes J Roderick P Rosenberg WM Evaluation of a prison outreach clinic for the diagnosis and prevention of hepatitis C: implications for the national strategy Gut 2003 52 10 1500 4 12970145 10.1136/gut.52.10.1500 PMC1773842

[B39] Sosman JM MacGowan RJ Margolis AD Eldridge E Flanigan T Vardaman J Fitzgerald C Kacanek D Binson D Seal DW Gaydos CA Screening for sexually transmitted diseases and hepatitis in 18-29-year-old men recently released from prison: feasibility and acceptability Int J STD AIDS 2005 16 2 117 22 15825246 10.1258/0956462053057594

[B40] Horne JA Clements AJ Drennan P Stein K Cramp ME Screening for hepatitis C virus in the Dartmoor prison population: an observational study Journal of Public Health 2004 26 4 372 375 15598857 10.1093/pubmed/fdh174

[B41] Martin NK Hickman M Miners A Hutchinson SJ Taylor A Vickerman P Cost-effectiveness of HCV case-finding for people who inject drugs via dried blood spot testing in specialist addiction services and prisons BMJ Open 2013 3 8 10.1136/bmjopen-2013-003153 PMC3752052 23943776

[B42] Khaw F Stobbart L Murtagh M ‘I just keep thinking I haven’t got it because I’m not yellow’: a qualitative study of the factors that influence the uptake of Hepatitis C testing by prisoners BioMed Central Public Health 2007 7 1 98 17555573 10.1186/1471-2458-7-98 PMC1906754

[B43] Chew KW Allen SA Taylor LE Rich JD Feller E Treatment Outcomes with Pegylated Interferon and Ribavirin for Male Prisoners with chronic Hepatitis C Journal of Clinical Gastroenterology 2009 43 7 686 691 19295448 10.1097/MCG.0b013e31818dd94c PMC2936234

[B44] Farley J Vasdev S Fischer B Haydon E Rehm J Farley TA Feasibility and outcome of HCV treatment in a Canadian Federal Prison Population American Journal of Public Health 2005 95 1737 1739 16131642 10.2105/AJPH.2004.056150 PMC1449428

[B45] Sterling RK Hofmann CM Luketic VA Sanyal AJ Contos MJ Miles AS Shiffman ML Treatment of chronic hepatitis C virus in the Virginia Department of Corrections: can compliance overcome racial differences to response American Journal of Gastroenterology 2004 99 866 872 15128352 10.1111/j.1572-0241.2004.30310.x

[B46] Allen SA Spaulding AC Osei AM Taylor LE Cabral AM Rich JD Treatment of chronic hepatitis C in a state correctional facility Ann Intern Med 2003 138 3 187 90 12558357 10.7326/0003-4819-138-3-200302040-00010

[B47] Maru DS Bruce RD Basu S Altice FL Clinical outcomes of hepatitis C treatment in a prison setting: feasibility and effectiveness for challenging treatment populations Clin Infect Dis 2008 47 7 952 61 18715156 10.1086/591707 PMC4847716

[B48] Strock P Mossong J Hawotte K Arendt V Access to treatment of hepatitis C in prison inmates Dig Dis Sci 2009 54 6 1325 30 18758958 10.1007/s10620-008-0483-8

[B49] Boonwaat L Haber PS Levy MH Lloyd AR Establishment of a successful assessment and treatment service for Australian prison inmates with chronic hepatitis C Med J 2010 192 9 496 500 10.5694/j.1326-5377.2010.tb03605.x 20438418

[B50] Bate JP Colman AJ Frost PJ Shaw DR Harley HA High prevalence of late relapse and reinfection in prisoners treated for chronic hepatitis C J Gastroenterol Hepatol 2010 25 1276 80 20594255 10.1111/j.1440-1746.2010.06295.x

[B51] Tan JA Joseph TA Saab T Treating Hepatitis C in the Prison Population Is Cost-Saving Hepatology 2008 1387 1395 http://onlinelibrary.wiley.com/doi/10.1002/hep.22509/pdf 18924228 10.1002/hep.22509

[B52] Hammett TM Making the case for health interventions in correctional facilities Journal of Urban Healt 2001 78 36 40 10.1093/jurban/78.2.236 PMC3456356 11419577

[B53] Spaulding AC Weinbaum CM Lau DT Sterling R Steff LB Margolis HB Hoofnagle JH A framework for management of hepatitis C in prisons Annals of Internal Medicine 2006 144 762 9 16702592 10.7326/0003-4819-144-10-200605160-00010

[B54] De Groot AS Stubblefield E Bick J Hepatitis C a correctional-public health opportunity Medscape Infectious Diseases 2001 3 1 14

[B55] Arora S Thornton K Jenkusky SM Parish B Scaletti JV Project ECHO: linking university specialists with rural and prison-based clinicians to improve care for people with chronic hepatitis C in New Mexico Public Health Rep 2007 122 Suppl 2 74 7 17542458 10.1177/00333549071220S214 PMC1831800

[B56] Klein SJ Wright LN Birkhead GS Mojica BA Klopf LC Klein LA Tanner EL Feldman IS Fraley EJ Promoting HCV treatment completion for prison inmates: New York State's hepatitis C continuity program Public Health Rep 2007 122 Suppl 2 83 8 17542460 10.1177/00333549071220S216 PMC1831802

[B57] Martin CK Hostetter JE Hagan JJ New opportunities for the management and therapy of hepatitis C in correctional settings Am J Public Health 2010 100 1 13 7 doi: 10.2105/AJPH.2008.147629 20007626 10.2105/AJPH.2008.147629 PMC2791263

[B58] Larrey D Salse A Ribard D Boutet O Hyrailles-Blanc V Niang B Pageaux GP Vaucher E Arpurt JP Boulay G Karlova N Daures JP Education by a nurse increases response of patients with chronic hepatitis C to therapy with peginterferon-alpha2a and ribavirin Clin Gastroenterol Hepatol 2011 9 9 781 5 21683161 10.1016/j.cgh.2011.05.022

[B59] Grogan A Timmins F Patient’s perceptions of information and support received from the nurse specialist during HCV treatment Journal of Clinical Nursing 2010 19 2869 2878 20846231 10.1111/j.1365-2702.2010.03239.x

[B60] Ehsani JP Vu T Karvelas M Exploring the need for hepatology nurses and allied health professionals in Victorian liver clinics Australian Health Review 2006 30 211 218 16646770 10.1071/ah060211

[B61] Leone NE The role of nursing in managing treatment-associated adverse effects in patients with hepatitis C Gastroenterology Nursing 2002 25 201 203 12394396 10.1097/00001610-200209000-00005

[B62] Nazareth S Piercey C Tibbet P Cheng W Innovative practice in the management of chronic Hepatitis C: introducing the nurse practitioner model Australian journal of advance nursing 2008 25 4 107 113

[B63] Stöver H Workshop results during 2nd International Symposium on Hepatitis care in substance users September 15-16, 2011 2012 Brussels/Belgium

[B64] ECDC EMCDDA Prevention and control of infectious diseases among people who inject drugs Guidance in brief 2011 http://www.emcdda.europa.eu/publications/ecdc-emcdda-guidance Accessed 30 March 2013

[B65] Weinbaum C Lyerla R Margolis HS Prevention and control of infections with hepatitis viruses in correctional settings. Morbidity and mortality weekly report. Recommendations and reports Centers for Disease Control 2003 52 1 36 12562146

[B66] Lines L Taking action to reduce injecting drug-related harms in prisons: the evidence of effectiveness of prison needle exchange in six countries International Journal of Prisoner Health 2005 1 49 64

[B67] Stöver H Nelles J Ten years of experience with needle and syringe exchange programmes in European Prisons International Journal of Drug Policy 2003 14 5/6 437 444

[B68] CDC Recommendations for prevention and control of hepatitis C virus (HCV) infection and HCV-related chronic disease MMWR, Recommendations and Reports 1998 47 RR-19 1 39 9790221

[B69] Broadhead RS Heckathorn DD Altice FL van Hulst Y Carbone M Friedland GH O'Connor PG Selwyn PA Increasing drug user’s adherence to IV treatment: results of a peer-driven intervention feasibility study Social Science and Medicine 2002 55 2 235 246 12144138 10.1016/s0277-9536(01)00167-8

[B70] Edlin BR Kresina TF Raymond DB Carden MR Gourevitch MN Rich JD Cheever LW Cargill VA Overcoming barriers to prevention, care and treatment of hepatitis C in illicit drug users Clinical Infectious Diseases 2005 40 5 276 285 10.1086/427441 PMC1510897 15768335

[B71] Hauser P Khosla J Aurora H Laurin J Kling MA Hill J Gulati M Thornton AJ Schultz RL Valentine AD Meyers CA Howell CD A prospective study of the incidence and open-label treatment of interferon-induced major depressive disorder in patients with hepatitis C Molecular Psychiatry 2002 7 9 942 947 12399946 10.1038/sj.mp.4001119

[B72] Belfiori B Ciliegi P Chiodera A Bacosi D Tosti A Baldelli F Francisci D Peginterferon plus ribavirin for chronic hepatitis C in opiate addicts on methadone/buprenorphine maintenance therapy Digestive and Liver Disease 2009 41 4 303 307 18938116 10.1016/j.dld.2008.08.009

[B73] Ferguson L Batey R Prisons, prisoners, and hepatitis C Journal of Gastroenterology and Hepatology 2014 25 7 1184 6 20594243 10.1111/j.1440-1746.2010.06357.x

